# Efficacy of Local Anesthesia for Radial Artery Puncture Pain: A Systematic Review and Network Meta-Analysis

**DOI:** 10.7759/cureus.64682

**Published:** 2024-07-16

**Authors:** Shunsuke Yasuo, Minoru Hayashi, Chiaki Suda, Yuki Kataoka, Shunsuke Taito, Eriya Imai, Kohei Sazanami

**Affiliations:** 1 Department of Emergency Medicine, Kyoto Katsura Hospital, Kyoto, JPN; 2 Department of Systematic Reviewers, Scientific Research WorkS Peer Support Group, Osaka, JPN; 3 Department of Emergency Medicine, Fukui Prefectural Hospital, Fukui, JPN; 4 Department of Public Health, Gunma University Graduate School of Medicine, Maebashi, JPN; 5 Department of Community Medicine, Section of Clinical Epidemiology, Kyoto University Graduate School of Medicine, Kyoto, JPN; 6 Department of Healthcare Epidemiology, Kyoto University School of Public Health, Kyoto, JPN; 7 Department of Internal Medicine, Kyoto Min-iren Asukai Hospital, Kyoto, JPN; 8 Department of Rehabilitation, Hiroshima University Hospital, Hiroshima, JPN; 9 Department of Anesthesia, Mitsui Memorial Hospital, Tokyo, JPN; 10 Department of Pharmacy, Kyoto Katsura Hospital, Kyoto, JPN

**Keywords:** systematic review, radial artery puncture, pain management, network meta-analysis, local anesthesia, analgesia

## Abstract

We performed a systematic review and network meta-analysis (NMA) to assist clinicians in determining the optimal patient-specific method of analgesia during radial artery puncture by comparing radial artery puncture procedural pain.

We included randomized controlled trials that assessed the prophylactic efficacy of local anesthesia for radial artery puncture-associated pain. We searched the Medical Literature Analysis and Retrieval System Online in January 2023, the Cochrane Central Register of Controlled Trials in January 2023, the Excerpta Medica Database in December 2022, the World Health Organization International Clinical Trials Platform Search Portal in January 2023, and ClinicalTrials.gov in January 2023. We synthesized the pain scores (0-100 scale) using the frequentist random-effects NMA model. We evaluated the confidence in each outcome using the CINeMA tool (https://cinema.ispm.unibe.ch/).

We conducted an NMA of 1,619 patients across 14 studies on pain scores during radial artery puncture-related procedures for 12 interventions. Compared with placebo, mepivacaine infiltration and lidocaine spray probably reduce pain (mean difference (MD): −47.67, 95% confidence interval (CI): −61.45 to −33.89, confidence rating (CR): moderate; MD: −27.38, 95% CI: −37.53 to −17.22, CR: moderate). Of the 32 studies included, none reported systemic adverse events, such as anaphylaxis or local anesthetic systemic toxicity, or severe local adverse events.

In conclusion, mepivacaine infiltration and lidocaine spray probably reduce the pain associated with radial artery puncture more than other local anesthesia.

## Introduction and background

Background

Radial arterial puncture (RAP) is a routine medical procedure frequently employed to obtain arterial blood samples for analysis in patients with impending respiratory failure or metabolic disorders, monitor critically ill patients, and facilitate contrast studies and endovascular therapy in patients with embolism or bleeding. Despite its ubiquity, RAP is associated with intense pain, and numerous studies have been aimed at identifying interventions to reduce discomfort [1]. A prior systematic review (SR) and meta-analysis (MA) highlighted the efficacy of local anesthesia categories such as infiltration anesthesia, cryotherapy, and topical anesthesia [1]. However, these categories vary regarding analgesic efficacy, onset time, and local anesthesia-associated pain for each local anesthesia technique. Recent randomized controlled trials (RCTs) have demonstrated the efficacy of lidocaine spray for topical anesthesia [2,3]. Despite these advancements, there remains a gap in comparative research on the efficacy of each local anesthesia technique to reduce RAP-associated intraprocedural pain.

Goals of this study

To address this gap, this SR and network meta-analysis (NMA) aimed to compare the effect sizes of multiple interventions for RAP-associated intraprocedural pain and provide an updated literature review. NMA is a statistical method that allows for both direct and indirect comparisons, even when treatment pairs have not been directly compared in the same trial [4-6].

## Review

Methods

Protocol and Registration

A pre-planned protocol for our SR was registered in the Open Science Framework (https://osf.io/eaxrv/?view_only=557f6c09bccf4f5a8e8230f0b974e54b) [7]. This NMA has been reported in accordance with the Preferred Reporting Items for Systematic Review and Meta-Analyses for Network Meta-Analyses (PRISMA-NMA) guidelines [8,9], as shown in Supplemental Material 1 (https://osf.io/eaxrv/?view_only=557f6c09bccf4f5a8e8230f0b974e54b). This SR and NMA were based on published data. As researchers did not access any information that could lead to identifying an individual patient, no ethical issue was raised in this research. Therefore, obtaining ethical approval and consent from participants was waived.

Eligibility Criteria

Type of studies: We included RCTs that assessed the prophylactic efficacy of local anesthesia for RAP-associated pain. We did not apply language or country restrictions. We included all papers, including published and unpublished articles, conference abstracts, and letters. We excluded quasi-randomized trials (such as those allocated by using alternate days of the week). We did not exclude studies based on the observation period or publication year.

Study participants: We included patients of both sexes, 16 years of age or older, before undergoing RAP. We included in RAP any technique that punctures the radial artery, such as arterial blood gas sampling (ABG), cannulation for continuous blood pressure monitoring or blood sampling (A-line), and introducer sheaths for angiography or endovascular treatment. Additionally, we included all local anesthesia methods and comparators. We distinguished between drugs according to the type and route of administration and placebo for local anesthetic infiltration (placebo infiltration) from those for other interventions due to the puncture pain caused by placebo infiltration [10]. For a eutectic mixture of local anesthetic creams of prilocaine and lidocaine (EMLA), if a single study involved multiple interventions based on the time from application to RAP, we chose the intervention closest to three hours [11]. We excluded patients who underwent other procedures, such as puncture of arteries or veins other than the radial artery. Furthermore, we excluded patients who were unable to communicate verbally because of disturbances in consciousness, cognitive impairment, or dysphasia.

Outcomes of Interest

The primary outcome was the pain score during RAP-related procedures, including local anesthesia. We converted the pain scores used in each study, such as the visual analog scale (VAS) and numeric rating scale (NRS), into a 0-100 scale (0: no pain, 100: worst pain), and then integrated them using the mean difference (MD) [12,13]. We classified infiltration anesthesia and cryotherapy as painful local anesthesia [14-17]. We utilized a combined pain score for the entire procedure, which included both local anesthesia and RAP. We employed a higher score in cases where a composite pain score was unavailable, although scores for individual components (pain from local anesthesia and pain from RAP) were present.

The secondary outcomes were the pain scores during RAP alone and all adverse events. If it was not specified whether the pain was due to RAP alone or any RAP-associated pain, we inquired with the author. If the author did not respond, we regarded the pain as due to RAP alone. We used the definition of adverse events set by the original authors and evaluated the incidence proportion of all adverse events during the follow-up period.

Search Strategy

We searched the Medical Literature Analysis and Retrieval System Online, the Cochrane Central Register of Controlled Trials, and the Excerpta Medica Database for studies published after January 1, 2020, as previous research has retrieved studies published through that date [1]. Furthermore, we searched the World Health Organization International Clinical Trials Platform Search Portal (ICTRP) and ClinicalTrials.gov for ongoing or unpublished trials. The details of the search strategy are provided in Supplemental Material 2 (https://osf.io/eaxrv/?view_only=557f6c09bccf4f5a8e8230f0b974e54b). We checked the reference lists of the studies, including international guidelines [18-20] and previous research [1], as well as the reference lists of eligible studies and articles citing eligible studies. We asked the authors of original studies for unpublished or additional data.

Study Selection and Data Extraction

Three independent reviewers (SY, MH, and CS) screened the titles and abstracts, assessed the eligibility based on the full texts, and performed independent data extraction from the included studies using a standardized data collection form. We contacted the original authors if relevant data were missing. Any disagreements were resolved through discussion, and a third reviewer acted as an arbiter (EI) if this failed.

Data Items

We extracted the following study characteristics: (1) methods: study design, author, language, year, country, and setting; (2) participants: number, sex, age, the reason for RAP, previous RAP, comorbidity, and inclusion/exclusion criteria (Allen test, allergy, cold-related reaction, etc.); (3) interventions: local anesthetic technique, needle size for RAP and infiltration, premedication, and healthcare professionals performing the procedure; and (4) outcomes: pain score (assessment tool and target) and adverse event.

Assessment of Risk of Bias

Three reviewers (SY, MH, and CS) independently evaluated the risk of bias (ROB) using the Risk of Bias 2 (The Cochrane Collaboration, London, England, UK) [21]. Disagreements between the two reviewers were discussed; if this failed, a third reviewer (EI) acted as an arbiter, if necessary.

Network Geometry

**Figure 1:**
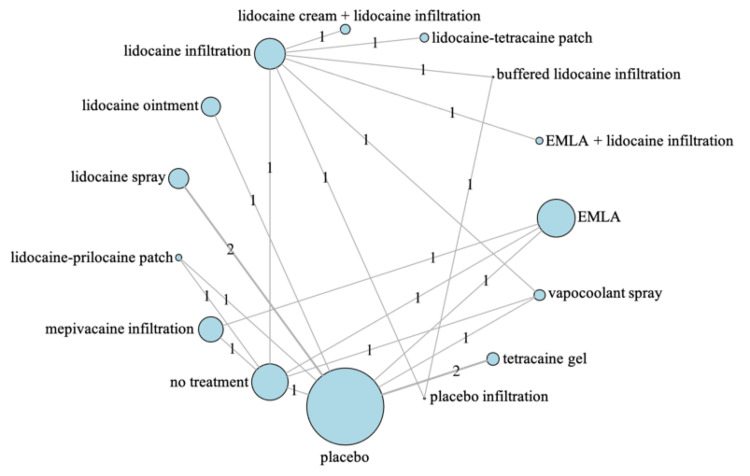
NMA for pain scores during radial artery puncture-related procedures The numbers above the lines represent the number of RCTs in direct comparisons. The size of the nodes reflects the number of direct comparisons in which the intervention took place. EMLA: eutectic mixture of local anesthetic cream of prilocaine and lidocaine, NMA: network meta-analysis, RCTs: randomized controlled trials Image Credit: Author

We demonstrated the network geometry (Figure 1). The numbers above the lines represent the number of RCTs in direct comparisons. The size of the nodes reflects the number of direct comparisons in which the intervention took place.

Data Synthesis and Statistical Analysis

We pooled the pain scores' MDs and 95% confidence intervals (CIs). We did not perform a quantitative analysis of adverse events because there were no reports of systemic or local severe adverse events, which we were intensely interested in, and few studies described minor local adverse events in detail. All the intervention groups that are relevant to this review were included. For continuous data, we did not impute missing data based on the recommendations of the Cochrane Handbook [22]. We performed a MA of the available data in the original studies. If the original studies did not report pain scores in each group as a combination of the mean and standard deviation, we calculated them based on the method described in the Cochrane Handbook [22]. The validity of these methods was analyzed using sensitivity analysis. We used group-level data. We used the normal likelihood for continuous outcomes. We synthesized the study effect sizes using a random-effects NMA model. We accounted for the correlations induced by multi-group studies by using multivariate distributions. The variance in the random-effects distribution (heterogeneity variance) was used to measure the extent of the across-study and within-comparison variability in treatment effects. To rank the treatments for each outcome, we used the surface under the cumulative ranking curve (SUCRA) [23]. We used MetaInsight for NMA [24].

Subgroup Analysis

To elucidate the influence of effect modifiers on the results, we performed subgroup analyses of the primary outcomes on the reason for RAP: ABG or A-line vs. introducer sheath for coronary angiography (CAG) or percutaneous coronary intervention (PCI).

Sensitivity Analysis

We performed the sensitivity analyses for the primary outcome by excluding studies that used imputation statistics to assess whether the review results were robust to the decisions made during the review process.

Assessment of Reporting Bias

We searched the clinical trial registry system (ClinicalTrials.gov and ICTRP) and performed an extensive literature search for unpublished trials. To assess the outcome reporting bias, we compared the outcomes defined in the trial protocols with those reported in the publications.

Assessment of the Confidence for Each Outcome

Two reviewers (SY and CS) evaluated the confidence for each primary outcome using the CINeMA tool [25,26]. The CINeMA framework includes the following domains: within-study bias, across-study bias, indirectness, imprecision, heterogeneity, and incoherence. For within-study bias and indirectness, CINeMA calculates the contribution of each study in each network estimate and combines these contributions with the study-specific evaluations (low, moderate, or high) to rate the relative effect for each comparison in the network. The domains of imprecision, heterogeneity, and incoherence use a pre-specified clinically important effect size to specify the margin of clinical equivalence between two interventions. Although no study has examined the minimal clinically important difference (MCID) for local anesthesia in RAP pain, based on several studies [27-29], we determined an MCID of 16 for the pain score on a 0-100 scale.

Results

Search Results

**Figure 2:**
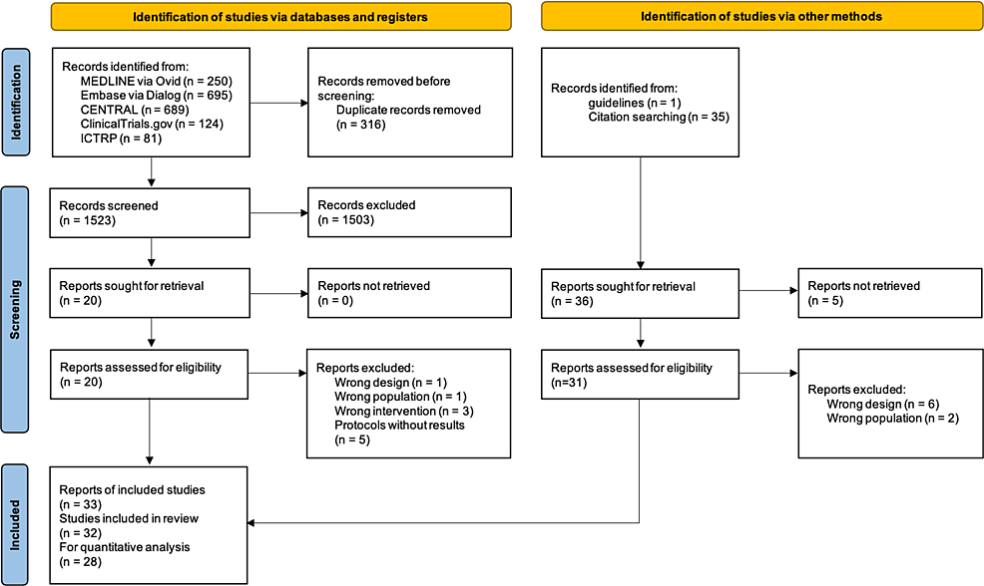
PRISMA 2020 flow diagram CENTRAL: Cochrane Central Register of Controlled Trials, Embase: Excerpta Medica Database, ICTRP: World Health Organization International Clinical Trials Platform Search Portal, MEDLINE: Medical Literature Analysis and Retrieval System Online, PRISMA: Preferred Reporting Items for Systematic Reviews and Meta-Analyses

A flow diagram of the study selection process is shown in Figure 2. After removing duplicates, we identified 1,523 records from the databases and registers by the January 30, 2023 search, and 36 records were identified from the citation searches and reference checks of the guidelines. We screened the full texts of 51 articles to identify the eligible studies, and we identified 32 eligible studies. We included a total of 28 trials in the qualitative analysis. More details of the excluded reports are provided in Supplemental Material 3 (https://osf.io/eaxrv/?view_only=557f6c09bccf4f5a8e8230f0b974e54b). We excluded four studies from the quantitative analysis because three did not describe the required data (number of participants in each group, mean or median of pain scores, and pain scores on a scale finer than 0-10) [30-32], and one involved interventions that could not be networked with other interventions [33].

Study Characteristics

The characteristics of the included studies are shown in Tables 1-2. We included 32 studies (n=3,568), in which 16 local anesthesia techniques (lidocaine infiltration, buffered lidocaine infiltration, mepivacaine infiltration, EMLA, tetracaine gel, lidocaine gel, lidocaine ointment, lidocaine jet injection, lidocaine-prilocaine patch, lidocaine-tetracaine patch, lidocaine iontophoresis, lidocaine spray, EMLA + lidocaine infiltration, lidocaine cream + lidocaine infiltration, vapocoolant spray, and ice pack) were performed [2,3,10,14-17,30-54]. The reasons for RAP included ABG in 22 studies [2,3,10,15-17,31-38,41,44,46,49,50,52-54], A-line in six studies [14,30,42,47,48,51], and CAG or PCI in four studies [39,40,43,45]. Only two studies used premedication with sedatives [30,48]. For ABG, A-line, and CAG or PCI (with 5-7 French introducer sheaths), 22-29, 17.5-20, and 18-20 gauge (G) needles, respectively, were used; 24-30 G needles were used for local anesthetic infiltration. The time from local anesthesia to RAP is 0-5 minutes for local anesthetic infiltration, cryotherapy, and lidocaine spray, whereas that for topical anesthesia other than lidocaine spray is longer (20-240 minutes).

**Table 1:** Characteristics of studies: baseline * We stated exclude if the study excluded Allen test-negative participants and unknown if it was not known to exclude Allen test-negative patients. We included in this category an assessment of ulnar artery patency other than the Allen test. † We described exclude if the study excluded patients with allergies to local anesthetics. In addition, we described it as exclude if the study excluded patients with cold-related reactions (e.g., Raynaud’s phenomenon) in studies where the intervention was cryotherapy. AMI: acute myocardial infarction, BA: bronchial asthma, CABG: coronary artery bypass grafting, CAD: cardiac artery disease, CAG: cardiac angiography, CHF: chronic heart failure, CKD: chronic kidney disease, COPD: chronic obstructive pulmonary disease, DL: dyslipidemia, DM: diabetes mellitus, ED: emergency department, EMLA: eutectic mixture of local anesthetic cream of prilocaine and lidocaine, HL: hyperlipidemia, HT: hypertension, ILD: interstitial lung disease, IP: interstitial pneumonia, MI: myocardial infarction, NR: no record, PCI: percutaneous coronary intervention, Q1/Q3: the 25 (lower)/75th (upper) percentiles of the data, RAP: radial artery puncture, SA: stable angina, SOB: shortness of breath, UA: unstable angina

Study name	Intervention/comparison	Setting	Sample size	Age		Male, %	Previous RAP	Co-morbidity	Allen test*	Allergy†
				Mean	SD					
Yıldız et al., 2021 [2]	Lidocaine spray	The ED	34	60	19/84	61.76	10	DM 7, HT 4, CHF 11, CKD 4, COPD/BA 8	Exclude	Exclude
	Placebo		33	58 median	24/86 min/max	66.67	13	DM 10, HT8, CHF 9, CKD 2, COPD/BA 10		
Gur and Tekin, 2021 [3]	Lidocaine spray	The ED of a tertiary hospital	72	49	10	61.11	37	Cases with peripheral neuropathy, DM, and methemoglobinemia were excluded	Unknown	Exclude
	Placebo		72	53	11	54.17	26			
Giner et al., 1996 [10]	Mepivacaine infiltration	Pulmonary function laboratory	70	61	12	66.19	111	NR	NR	NR
	Placebo infiltration		70							
	No treatment		70							
Ruetzler et al., 2012 [14]	Lidocaine infiltration	Elective valve replacement or CABG	45	69	59/74	73.33	NR	Type of surgery: CABG 18, valve 22, CABG + valve 5	Exclude	Exclude
	Lidocaine-tetracaine patch		45	68 median	54/75 Q1/Q3	82.22		Type of surgery: CABG 17, valve 20, CABG + valve 8		
Farahmand et al., 2017 [15]	Vapocoolant spray	The ED	40	56.5	20/76	55	NR	NR	Exclude	Exclude
	Placebo		40	48.5	19/75 min/max	47.50				
Pagnucci et al., 2020 [16]	Mepivacaine infiltration	The ED of a teaching hospital	133	57	NR	63.91	NR	NR	Unknown	Exclude
	Ice pack		134	59	NR	60.45				
	EMLA		133	55	NR	54.89				
	No treatment		133	57 median	NR	52.63				
Mahto et al., 2016 [17]	Ice pack	Medical ICU of the medical college hospital	40	52.6	16	71.7	NR	NR	NR	NR
	No treatment		40	54.2	18	65.5				
Olday et al., 2002 [30]	Lidocaine infiltration	Elective cardiac surgery	99	NR	NR	NR	NR	NR	Exclude	Exclude
	Tetracaine gel									
Godoy Mayoral et al., 2010 [31]	EMLA	The oxygen therapy office	23	76	10.98	64.71	NR	NR	NR	NR
	Placebo		28							
Lightowler and Elliott, 1997 [32]	Lidocaine infiltration	The university hospital respiratory unit	33	NR	NR	NR	NR	NR	NR	Exclude
	Placebo infiltration		34							
	No treatment		34							
Hajiseyedjavady et al., 2012 [33]	Lidocaine jet injection	The ED	21	52	19/85	71.43	NR	NR	NR	Exclude
	Lidocaine gel		21	60 median	21/89 min/max	47.62				
Beaumont et al., 2021 [34]	EMLA	Hospitalized in or referred to the pneumology department	67	64	12	61.19	53	NR	Exclude	Exclude
	Placebo		69	65	12	72.46	52			
Dhami et al., 2020 [35]	Vapocoolant spray	Admitted to the pulmonary high-dependency unit of a tertiary care hospital	30	50	9	80	21	13	Exclude	Exclude
	Ice pack		30	45	10	83.33	21	17 details unknown		
Haynes, 2015 [36]	Ice pack	The hospital-based pulmonary function laboratory	40	64.9	13.3	52.50	20	NR	NR	Exclude
	No treatment		40	64.4	15.3	45	20			
Wade et al., 2015 [37]	Lidocaine infiltration	The ED	20	53.2	21.5	65	NR	NR	Exclude	NR
	No treatment		21	54.8	20.5	57.14				
Khalil, 2017 [38]	Ice pack	Medical critical care and emergency units	50	56	2.1	62	10	NR	Exclude	Exclude
	No treatment		50	54	2.1	54	11			
Youn et al., 2011 [39]	EMLA + lidocaine infiltration	Referred for CAG	38	55	9	60.53	NR	HT 22, DM 8, DL 5, MI 1, coronary intervention 2	NR	NR
	Lidocaine infiltration		38	53	8	63.16		HT 17, DM 9, DL 12, MI 2, coronary intervention 6		
Tatlı et al., 2018 [40]	Lidocaine cream + lidocaine infiltration	Presented to the university hospital with an indication for CAG	52	60.5	9.4	69.23	NR	HT 29, DM 23, HL 9, known CAD 12	NR	Exclude
	Lidocaine infiltration		52	60.4	9.7	69.23		HT 32, DM 16, HL 13, known CAD 13		
Bastami et al., 2015 [41]	Ice pack	Admitted to a public educational center (the emergency ward)	31	61.25	12.61	58.06	18	NR	Exclude	Exclude
	No treatment		30	62.2	14.99	53.33	16			
Sherwin et al., 2003 [42]	Lidocaine infiltration	Vascular surgery requiring intraarterial pressure monitoring	15	70.7	8.5	80	NR	NR	NR	NR
	Lidocaine iontophoresis		15	73	6.6	86.67				
Kim et al., 2017 [43]	EMLA + lidocaine infiltration	Elective CAG or PCI	45	61	9	66.67	NR	SA 17, UA 17, AMI 4	Exclude	Exclude
	Lidocaine infiltration		98	60	10	59.18		SA 31, UA 33, AMI 8		
Matheson et al., 2014 [44]	Lidocaine infiltration	Hospitalized	10	NR	NR	NR	NR	NR	Exclude	Exclude
	Buffered lidocaine infiltration		10							
	Placebo infiltration		10							
Latsios et al., 2017 [45]	Lidocaine infiltration	Referred with the suspicion of CAD elective CAG	219	64.76	10.7	74.43	NR	HT133, DL 107, DM 42	Exclude	Exclude
	EMLA		225	65.74	11.5	72		HT135, DL 110, DM 54		
Aaron et al., 2003 [46]	Tetracaine gel	Referred to the pulmonary function laboratory	24	60	12	66.67	7	BA 3, COPD 5, lung cancer 12, ILD 0	Unknown	Exclude
	Placebo		26	61	15	69.23	3	BA 5, COPD 7, lung cancer 13, ILD 1		
Smith et al., 1990 [47]	EMLA + lidocaine infiltration	Neurosurgical patients who required arterial cannulation	10	48.3	22/69 min/max	NR	NR	NR	NR	NR
	EMLA		10							
	Lidocaine infiltration		10							
Russell et al., 1988 [48]	Lidocaine infiltration	Elective cardiac surgery	20	59	9	50	NR	NR	NR	Exclude
	EMLA		20	62	9	50				
Tran et al., 2002 [49]	Tetracaine gel	The respiratory function laboratory, the respiratory outpatients, the oxygen clinic, and the respiratory ward at a major teaching hospital	42	66.4	13.3	59.52	32	NR	Exclude	Exclude
	Placebo		39	64.3	15.2	66.67	29			
France et al., 2008 [50]	Lidocaine infiltration	Two urban EDs	20	62	25/92	60	NR	SOB 2, COPD 11, BA 0, possible PE 4, metabolic 1	NR	Exclude
	Vapocoolant spray		18	57	29/83	55.56		SOB 3, COPD 4, BA 0, possible PE 8, metabolic 2		
	No treatment		21	55	22/81 min/max	52.38		SOB 1, COPD 6, BA 2, possible PE 9, metabolic 2		
Rüsch et al., 2017 [51]	Lidocaine infiltration	elective cardiac surgery or carotid endarterectomy	69	68	8	46.38	NR	NR	Exclude	Exclude
	Vapocoolant spray		74	67	10	40.54				
Micu et al., 2006 [52]	Lidocaine–prilocaine patch	Lung function laboratory	34	60	2	67.65	NR	NR	NR	NR
	Placebo		31	56	3	58.06				
	No treatment		38	53	2	76.32				
Cortés-Telles et al., 2012 [53]	Lidocaine ointment	Pulmonary function laboratory	102	57	18	50	NR	Obstructive diseases (including COPD, BA, and bronchiectasis) 42%, IP 30%	Exclude	Exclude
	Placebo		98	56	18	44.9				
Giner et al., 1997 [54]	Mepivacaine infiltration	NR	30	NR	NR	NR	NR	NR	NR	NR
	No treatment		30							

**Table 2:** Characteristics of studies: intervention * We noted that "immediately" or "subsequently" is less than one minute. ABG: arterial blood gas sampling, A-line: arterial line for cannulation for continuous blood pressure monitoring or blood sampling, CAG: cardiac angiography, ED: emergency department, EMLA: eutectic mixture of local anesthetic cream of prilocaine and lidocaine, Fr: French, G: gauze, ICU: intensive care unit, NR: no record, PCI: percutaneous coronary intervention, RAP: radial artery puncture, TRA: transradial coronary angiography, -: not applicable

Study name	Intervention/comparison	Details of intervention	Reason for RAP	Pre-medication	Needle gauge (LA infiltration)	Time from LA to RAP, min*	Healthcare professionals
Yıldız et al., 2021 [2]	Lidocaine spray	10%, spraying six times	ABG	NR	22	5	Emergency medicine residents
	Placebo	-				-	
Gur and Tekin, 2021 [3]	Lidocaine spray	10%, spraying six times (each spray contained 0.1 ml)	ABG	NR	25	4	The same physician in the ED
	Placebo	-				-	
Giner et al., 1996 [10]	Mepivacaine infiltration	1%, 0.2 mL	ABG	NR	22 (27.5)	<1	NR
	Placebo infiltration	Saline, 0.2 mL					
	No treatment	-					
Ruetzler et al., 2012 [14]	Lidocaine infiltration	1%, 0.5 mL	A-line	NR	20 (NR)	3	An independent researcher
	Lidocaine-tetracaine patch	70 mg each of lidocaine and tetracaine				20	
Farahmand et al., 2017 [15]	Vapocoolant spray	5 seconds	ABG	NR	23	<1	An experienced 2nd year ED resident
	Placebo	-				-	
Pagnucci et al., 2020 [16]	Mepivacaine infiltration	1%, 1 mL	ABG	NR	22 (30)	10 seconds	An expert nurse, who was also certified in this procedure
	Ice pack	A plastic bag containing 200 grams of ice				3	
	EMLA	5%, 2 g				60	
	No treatment	-				-	
Mahto et al., 2016 [17]	Ice pack	NR	ABG	NR	23	3	The respiratory therapist
	No treatment	-				-	
Olday et al., 2002 [30]	Lidocaine infiltration	2%, 0.5-0.7 mL	A-line	Lorazepam 1-3 mg	20 (25)	1	An experienced registrar or consultant anaesthetist
	Tetracaine gel	4%				60	
Godoy Mayoral et al., 2010 [31]	EMLA	1 mL	ABG	NR	23 G or 0.57 mm in diameter	30	The nurses of the laboratory
	Placebo	-					
Lightowler and Elliott, 1997 [32]	Lidocaine infiltration	2%, 0.5 mL	ABG	None	29 (NR)	2	Senior house officers in respiratory medicine
	Placebo infiltration	Normal saline, 0.5 mL				-	
	No treatment					-	
Hajiseyedjavady et al., 2012 [33]	Lidocaine jet injection	2%, 0.2 mL	ABG	NR	29	5	Experienced postgraduate year–3 ED residents
	Lidocaine gel	2%, 1 mL					
Beaumont et al., 2021 [34]	EMLA	5%, 2 g	ABG	NR	23	120	Nurses in the respiratory care unit
	Placebo	-				-	
Dhami et al., 2020 [35]	Vapocoolant spray	3 seconds	ABG	NR	23	<1	The primary investigator
	Ice pack	-				3	
Haynes, 2015 [36]	Ice pack	A small plastic bag filled with 12 oz of crushed ice	ABG	NR	23	3	The principle investigator
	No treatment	-				-	
Wade et al., 2015 [37]	Lidocaine infiltration	1%, 1 mL	ABG	NR	22 or 25 (30)	10 seconds	The 1st author
	No treatment	-				-	
Khalil, 2017 [38]	Ice pack	NR	ABG	NR	25	10	The researcher
	No treatment	-				-	
Youn et al., 2011 [39]	EMLA + lidocaine infiltration	EMLA: 5%, 2.5 g lidocaine infiltration: 2%, 0.6 mL	CAG	NR	20 G→5Fr sheath (26)	60-180	The operator experienced with TRA in 1,000 or more cases in one year
	Lidocaine infiltration					NR	
Tatlı et al., 2018 [40]	Lidocaine cream + lidocaine infiltration	lidocaine cream: 5% lidocaine infiltration: 1%, 1 mL	CAG or PCI	NR	20 G→5Fr sheath (30)	30+3	An experienced cardiologist
	Lidocaine infiltration					3	
Bastami et al., 2015 [41]	Ice pack	NR	ABG	NR	25	5	One researcher experienced with the procedure to maintain consistency
	No treatment	-				-	
Sherwin et al., 2003 [42]	Lidocaine infiltration	1%, 1 mL	A-line	None	20 (25)	1	An investigator who was blinded to the method of analgesia used
	Lidocaine iontophoresis	4%, 4 ml				10	
Kim et al., 2017 [43]	EMLA + lidocaine infiltration	5%, 2.5 g	CAG or PCI	None	20 G→5-7Fr (24)	180-240	Internship medical doctors
	Lidocaine infiltration	0.6 mL				NR	
Matheson et al., 2014 [44]	Lidocaine infiltration	1%, 0.7 ml	ABG	NR	NR (NR)	NR	One researcher who had 12 years of experience as a critical care nurse and routinely draws ABGs on patients in the ICUs
	Buffered lidocaine infiltration	1%, 0.7 ml					
	Placebo infiltration	Bacteriostatic saline, 0.7 ml					
Latsios et al., 2017 [45]	Lidocaine infiltration	2%, 1-2 mL	CAG	None (IV sedation)	20 G→6Fr sheath (26)	1	Experienced and radial dedicated interventional cardiologists
	EMLA	5%, 2.5 g				30	
Aaron et al., 2003 [46]	Tetracaine gel	4％, 1 g	ABG	None	23	45	The respiratory therapist
	Placebo	-					
Smith et al., 1990 [47]	EMLA + lidocaine infiltration	EMLA: 5% lidocaine infiltration: 1%, 0.2-0.3 mL	A-line	None	20 (25)	60+2	One of the authors
	EMLA					60	
	Lidocaine infiltration					2	
Russell et al., 1988 [48]	Lidocaine infiltration	2%, 1-2 mL	A-line	Diazepam 5-10 mg orally	17.5 (25)	2	One of four senior clinicians
	EMLA	5%, 2.5 g				90	
Tran et al., 2002 [49]	Tetracaine gel	4%	ABG	NR	25	30	Respiratory scientists or hospital medical officers experienced with the procedure
	Placebo	-				-	
France et al., 2008 [50]	Lidocaine infiltration	2%, 0.5 mL	ABG	NR	23 (25)	5	ED doctors (29 different doctors)
	Vapocoolant spray	Until the skin becomes "frosted"				<1	
	No treatment	-				-	
Rüsch et al., 2017 [51]	Lidocaine infiltration	2%, 0.5 mL	A-line	NR	20 (27)	<1	The same physician who was in the fourth year of his anesthesia residency
	Vapocoolant spray	Approximately 2 to 3 seconds until the skin surface turns white in color				<1	
Micu et al., 2006 [52]	Lidocaine–prilocaine patch	NR	ABG	NR	26	60	One of the four nurses
	Placebo	-				-	
	No treatment	-				-	
Cortés-Telles et al., 2012 [53]	Lidocaine ointment	5%, 2 g (1 g equals 250 mg of lidocaine)	ABG	NR	27	30	A respiratory technician
	Placebo	-				-	
Giner et al., 1997 [54]	Mepivacaine infiltration	NR	ABG	NR	22 (27.5)	NR	NR
	No treatment	-			25	-	

Primary Outcome

**Figure 3:**
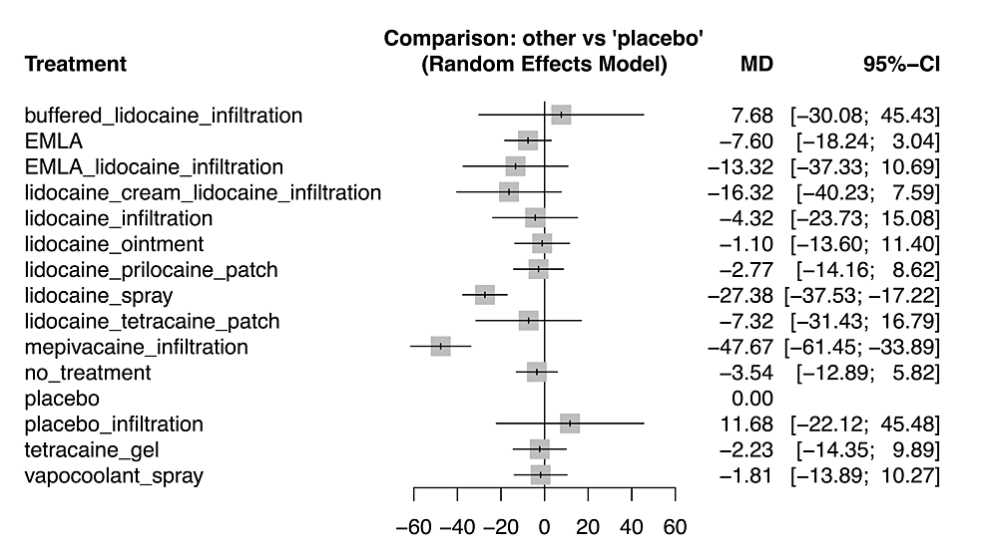
Forest plot for all interventions compared with placebo in pain scores during radial artery puncture-related procedure We conducted an NMA with 1,619 patients from 14 studies on pain scores during radial artery puncture-related procedures for 12 interventions [2,3,14–16,34,39,40,44,46,49,50,52,53]. EMLA: eutectic mixture of local anesthetic cream of prilocaine and lidocaine, NMA: network meta-analysis, MD: mean difference, CI: confidence interval

Pain scores during RAP-related procedures: We conducted an NMA with 1,619 patients from 14 studies on pain scores during RAP-related procedures for 12 interventions (Figure 1) [2,3,14-16,34,39,40,44,46,49,50,52,53]. Supplemental Materials 4, 5, and 6 display each study's results and ROB (https://osf.io/eaxrv/?view_only=557f6c09bccf4f5a8e8230f0b974e54b). A forest plot comparing each intervention with the placebo is shown in Figure 3, and the confidence ratings (CR) for the results are presented in Table 3. Compared with placebo, mepivacaine infiltration and lidocaine spray probably reduce RAP-associated pain (MD: -47.67, 95% CI: -61.45 to -33.89, CR: moderate; MD: -27.38, 95% CI: -37.53 to -17.22, CR: moderate). We present the ranking table and league table details in Table 4 and Supplemental Material 7 (https://osf.io/eaxrv/?view_only=557f6c09bccf4f5a8e8230f0b974e54b), respectively.

**Table 3:** The confidence for pain scores during radial artery puncture-related procedures assessed by the CINeMA tool EMLA: eutectic mixture of local anesthetic cream of prilocaine and lidocaine, CR: confidence rating

Interventions vs. placebo	Within-study bias	Reporting bias	Indirectness	Imprecision	Heterogeneity	Incoherence	CR
EMLA	Major concerns	Low risk	No concerns	Some concerns	Some concerns	No concerns	Very low
Lidocaine ointment	Some concerns	Low risk	No concerns	No concerns	Major concerns	No concerns	Low
Lidocaine spray	Some concerns	Low risk	No concerns	No concerns	No concerns	No concerns	Moderate
Lidocaine-prilocaine patch	Some concerns	Low risk	No concerns	No concerns	Major concerns	No concerns	Low
No treatment	Some concerns	Low risk	No concerns	No concerns	Major concerns	No concerns	Low
Tetracaine gel	Some concerns	Low risk	No concerns	No concerns	Major concerns	No concerns	Low
Vapocoolant spray	No concerns	Low risk	Some concerns	No concerns	Major concerns	No concerns	Low
EMLA + lidocaine infiltration	Some concerns	Low risk	Some concerns	Some concerns	Some concerns	No concerns	Low
Buffered lidocaine infiltration	Some concerns	Low risk	No concerns	Major concerns	No concerns	No concerns	Low
Lidocaine-tetracaine patch	Some concerns	Low risk	Some concerns	Major concerns	No concerns	No concerns	Very low
Lidocaine cream + lidocaine infiltration	Some concerns	Low risk	No concerns	Some concerns	Some concerns	No concerns	Moderate
Lidocaine infiltration	Some concerns	Low risk	Some concerns	Some concerns	Some concerns	No concerns	Low
Mepivacaine infiltration	Some concerns	Low risk	Some concerns	No concerns	No concerns	No concerns	Moderate
Placebo infiltration	Some concerns	Low risk	No concerns	Major concerns	No concerns	No concerns	Low

**Table 4:** Ranking table for all interventions compared with placebo in pain scores during radial artery puncture-related procedures * The SUCRA quantifies the overall ranking: the closer the SUCRA value is to 100, the more likely the treatment is higher ranked; the closer the SUCRA value is to 0, the more likely the treatment is lower ranked. EMLA: eutectic mixture of local anesthetic cream of prilocaine and lidocaine, SUCRA: surface under the cumulative ranking curve

Rank	Treatment	SUCRA*
1	Mepivacaine infiltration	97.68
2	Lidocaine spray	85.31
3	Lidocaine cream + lidocaine infiltration	70.31
4	EMLA + lidocaine infiltration	64.84
5	EMLA	55.83
6	Lidocaine-tetracaine patch	51.76
7	Lidocaine infiltration	44.34
8	Lidocaine-prilocaine patch	42.03
9	Tetracaine gel	40.23
10	Vapocoolant spray	38.68
11	Lidocaine ointment	37.70
12	Buffered lidocaine infiltration	26.02

Secondary Outcomes

**Figure 4:**
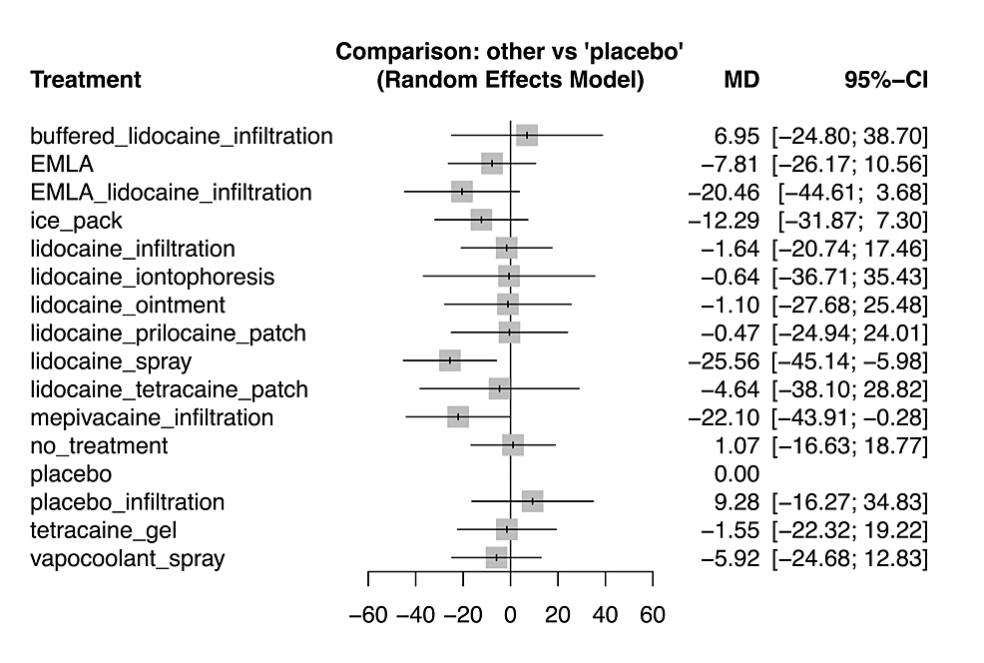
Forest plot for all interventions compared with placebo in pain scores during radial artery puncture alone We conducted an NMA with 3,171 patients from 27 studies on pain scores during radial artery puncture alone for 13 interventions [2,3,10,14–17,34–39,41–54]. EMLA: eutectic mixture of local anesthetic cream of prilocaine and lidocaine, NMA: network meta-analysis, MD: mean difference, CI: confidence interval

Pain scores during RAP alone: We conducted an NMA with 3,171 patients from 27 studies on pain scores during RAP alone for 13 interventions [2,3,10,14-17,34-39,41-54]. Supplemental Materials 4, 5, and 6 display each study's results and ROB (https://osf.io/eaxrv/?view_only=557f6c09bccf4f5a8e8230f0b974e54b). A forest plot comparing each intervention with the placebo is shown in Figure 4. Similar trends to the primary outcome were observed, but no intervention significantly reduced pain as compared with the placebo. Among interventions evaluated only as a secondary outcome (ice pack and lidocaine iontophoresis), ice packs may reduce pain (MD: −12.29, 95% CI: −31.87 to 7.30).

Adverse events: Of the 32 studies included, none reported systemic adverse events such as anaphylaxis or local anesthetic systemic toxicity and severe local adverse events [2,3,10,14-17,30-54]. Mild adverse events, such as erythema or hematoma, have been reported and are detailed in Table 5. Many studies excluded patients with allergies to local anesthetics or those with cold-related reactions (e.g., Raynaud’s phenomenon) (Table 1).

**Table 5:** Adverse events * The denominator could increase by 2 in which case it would be 4.55% (2/44). † Mild blanching was observed in eight patients in the EMLA groups. EMLA groups included the EMLA-only group, EMLA + placebo infiltration group, and EMLA + lidocaine infiltration group. EMLA: eutectic mixture of local anesthetic cream of prilocaine and lidocaine, NR: no record, RA: radial artery, -: not applicable

Study name	Intervention/comparison	Adverse event, % (n)	
Yıldız et al., 2021 [2]	Lidocaine spray	None	-
	Placebo	None	-
Gur and Tekin, 2021 [3]	Lidocaine spray	None	-
	Placebo	None	-
Giner, 1996 [10]	Mepivacaine infiltration	NR	-
	Placebo infiltration	NR	-
	No treatment	NR	-
Ruetzler et al., 2012 [14]	Lidocaine infiltration	Erythema	4.44 (2)
	Lidocaine-tetracaine patch	Erythema	6.67 (3)
Farahmand et al., 2017 [15]	Vapocoolant spray	Numbness	Some
	Placebo	NR	-
Pagnucci et al., 2020 [16]	Mepivacaine infiltration	NR	-
	Ice pack	NR	-
	EMLA	NR	-
	No treatment	NR	-
Mahto et al., 2016 [17]	Ice pack	Not tolerate ice application	4.76 (2)*
	No treatment	NR	-
Olday et al., 2002 [30]	Lidocaine infiltration	NR	-
	Tetracaine gel	Erythema	Common
Godoy Mayoral et al., 2010 [31]	EMLA	NR	-
	Placebo	NR	-
Lightowler and Elliott, 1997 [32]	Lidocaine infiltration	None	-
	Placebo infiltration	None	-
	No treatment	None	-
Hajiseyedjavady et al., 2012 [33]	Lidocaine jet injection	None	-
	Lidocaine gel	NR	-
Beaumont et al., 2021 [34]	EMLA	No significant between-group differences (details unknown)	-
	Placebo		
Dhami et al., 2020 [35]	Vapocoolant spray	Hematoma	6.67 (2)
	Ice pack	Hematoma	33.33 (10)
Haynes, 2015 [36]	Ice pack	Not tolerate ice application	7.5 (3)
	No treatment	NR	-
Wade et al., 2015 [37]	Lidocaine infiltration	None	-
	No treatment	None	-
Khalil, 2017 [38]	Ice pack	NR	-
	No treatment	NR	-
Youn et al., 2011 [39]	EMLA + lidocaine infiltration	NR	-
	Lidocaine infiltration	NR	-
Tatlı et al., 2018 [40]	Lidocaine cream + lidocaine infiltration	Complications (details unknown)	13.46 (7)
	Lidocaine infiltration	Complications (details unknown)	28.85 (15)
Bastami et al., 2015 [41]	Ice pack	NR	-
	No treatment	NR	-
Sherwin et al., 2003 [42]	Lidocaine infiltration	NR	-
	Lidocaine iontophoresis	Slight erythema	NR
Kim et al., 2017 [43]	EMLA + lidocaine infiltration	Erythema	6.6 (3)
	Lidocaine infiltration	NR	-
Matheson et al., 2014 [44]	Lidocaine infiltration	NR	-
	Buffered lidocaine infiltration	NR	-
	Placebo infiltration	NR	-
Latsios et al., 2017 [45]	Lidocaine infiltration	None	-
	EMLA	None	-
Aaron et al., 2003 [46]	Tetracaine gel	Redness	4.17 (1)
		Swelling	4.17 (1)
		Itching	4.17 (1)
	Placebo	Redness	3.85 (1)
		Swelling	3.85 (1)
		Bruising	11.54 (3)
Smith et al., 1990 [47]	EMLA + lidocaine infiltration	Mild blanching	26.67 (8)†
		Erythema	NR
	EMLA	Mild blanching	NR
		Erythema	NR
	Lidocaine infiltration	Mild blanching	20 (2)
		Erythema	NR
Russell et al., 1988 [48]	Lidocaine infiltration	NR	-
	EMLA	Pallor and slight edema	Common
Tran et al., 2002 [49]	Tetracaine gel	Self-limiting itchiness	2.38 (1)
	Placebo	Tingling feeling	2.56 (1)
		A local sensation of swelling	2.56 (1)
		A transient fainting feeling	2.56 (1)
France et al., 2008 [50]	Lidocaine infiltration	NR	-
	Vapocoolant spray	NR	-
	No treatment	NR	-
Rüsch et al., 2017 [51]	Lidocaine infiltration	Local complications (details unknown)	8.75 (7)
	Vapocoolant spray	Local complications (details unknown)	5 (4)
Micu et al., 2006 [52]	Lidocaine–prilocaine patch	NR	-
	Placebo	NR	-
	No treatment	NR	-
Cortés-Telles et al., 2012 [53]	Lidocaine ointment	None	-
	Placebo	None	-
Giner et al., 1997 [54]	Mepivacaine infiltration	NR	-
	No treatment	NR	-

Subgroup Analysis

**Figure 5:**
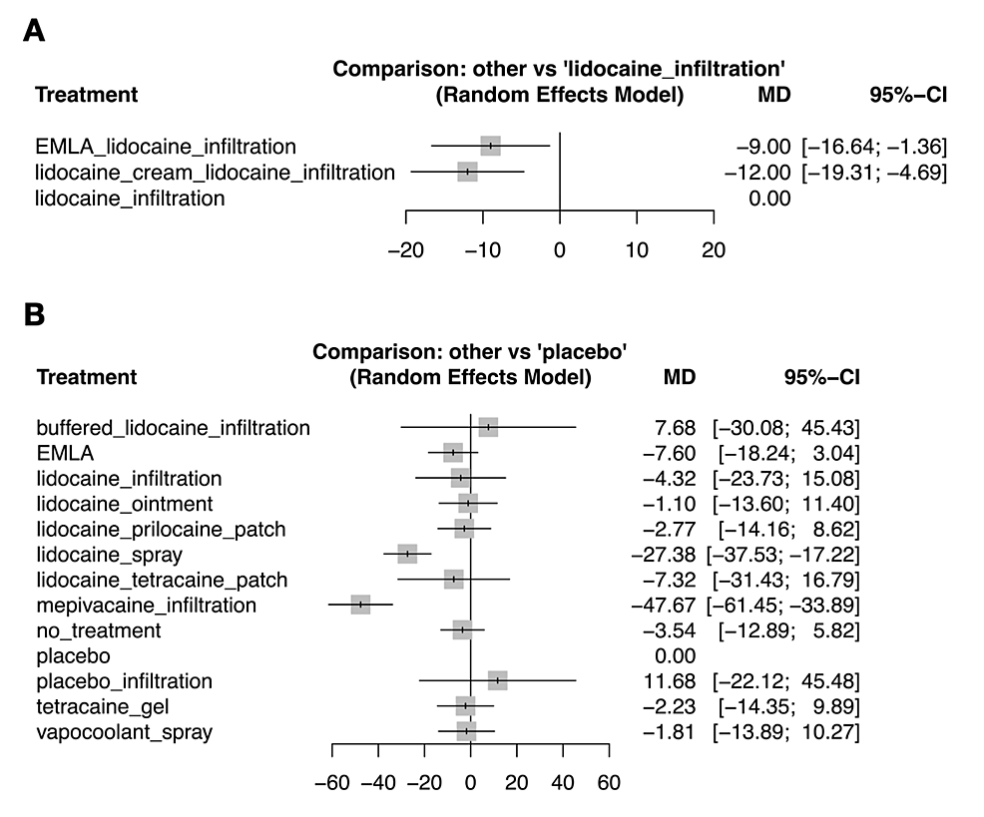
Forest plot of subgroup analysis in pain scores during radial artery puncture-related procedures A: Radial artery puncture for CAG or PCI. B: Radial artery puncture for ABG or cannulation for continuous blood pressure monitoring or blood sampling. We conducted a subgroup analysis of the radial artery puncture-associated pain scores between radial artery puncture for CAG or PCI [39,40] vs. ABG or cannulation for continuous blood pressure monitoring or blood sampling [2,3,14–16,34,44,46,49,50,52,53]. EMLA: eutectic mixture of local anesthetic cream of prilocaine and lidocaine, ABG: arterial blood gas sampling, CAG: coronary angiography, PCI: percutaneous coronary intervention, MD: mean difference, CI: confidence interval

We conducted a subgroup analysis of the RAP-associated pain scores between RAP for CAG or PCI vs. ABG or A-line (Figure 5). In RAP for CAG or PCI, EMLA or lidocaine cream plus lidocaine infiltration may reduce pain associated with RAP compared with lidocaine infiltration alone (MD: −9.00, 95% CI: −16.64 to −1.36, MD: −12.00, 95% CI: −19.31 to −4.69) [39,40].

Sensitivity Analysis

**Figure 6:**
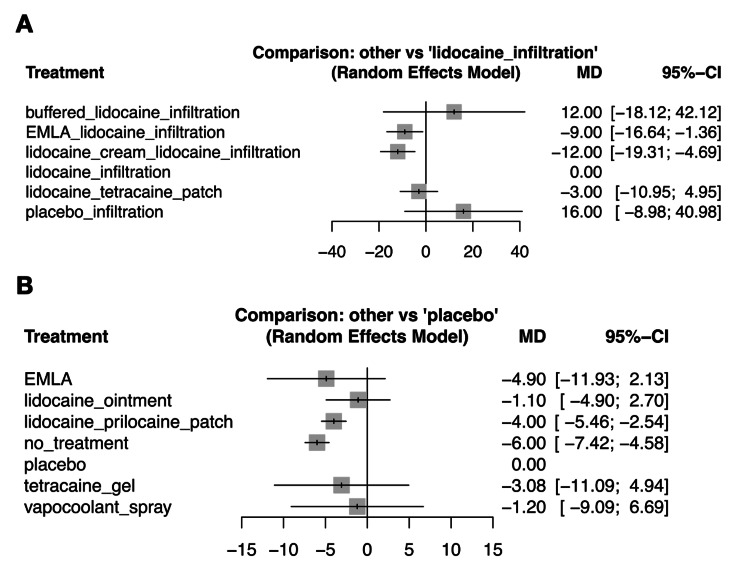
Forest plot of sensitivity analysis in pain scores during radial artery puncture-related procedures: exclusion of studies using imputed statistics A: Interventions forming a network connected by lidocaine infiltration. B: Interventions forming a network connected by placebo. We undertook a sensitivity analysis of radial artery puncture-associated pain scores by excluding studies that used imputed statistics [15,34,39,40,44,46,49,52,53]. EMLA: eutectic mixture of local anesthetic cream of prilocaine and lidocaine, MD: mean difference, CI: confidence interval

We undertook a sensitivity analysis of RAP-associated pain scores by excluding studies that used imputed statistics (Figure 6) [15,34,39,40,44,46,49,52,53]. However, the results did not significantly differ from the primary outcome results.

Discussion

Summary of Results

This SR and NMA included 14 RCTs that compared the efficacy and safety of all interventions on pain scores during RAP-related procedures. Mepivacaine infiltration and lidocaine spray were probably more effective than other local anesthesias. No study reported systemic adverse events such as anaphylaxis, local anesthetic systemic toxicity, or severe local adverse events.

Comparison With the Previous Studies

Our study highlights the efficacy of lidocaine spray, which is a form of topical anesthesia that has not been considered in previous research. The advantage of lidocaine spray is its more rapid effect (within four to five minutes) than other topical anesthetics [55-57]. Physicians can utilize lidocaine spray in situations with time constraints, such as EDs.

Despite using the same route of administration and drugs with similar characteristics [58], our study demonstrated the efficacy of mepivacaine infiltration but not lidocaine infiltration. Although the apparent cause of this phenomenon remains unknown, we can attribute it to several suggested factors.

Firstly, the observed variation may have been influenced by the vasodilatory effects of these drugs. Mepivacaine demonstrated a milder vasodilatory effect than lidocaine [59]. In a SR that compared mepivacaine and lidocaine for local anesthesia in dentistry, mepivacaine with adrenaline was found to be more effective than lidocaine with adrenaline. Conversely, when mepivacaine alone was compared with lidocaine with adrenaline, lidocaine with adrenaline was found to be more effective [60]. This result indicates the potential influence of vasodilatory or vasoconstrictive effects on the analgesic efficacy of mepivacaine and lidocaine. A previous study has suggested that subcutaneous injection-induced pain may intensify in the presence of bleeding [61]. If the milder vasodilatory effect of mepivacaine compared to that of lidocaine contributes to a reduction of RAP-linked bleeding, it may consequently alleviate the associated pain.

Secondly, the observed variation might have been influenced by the size of the needle used for subcutaneous injection. Using thinner needles for subcutaneous injections reduces patient pain scores and minimizes the percentage of patients experiencing pain [61-63]. Moreover, employing smaller needles for subcutaneous injections may not only diminish injection pain but also alleviate the pain from RAP alone because of the potential influence of anxiety about future pain and past pain experiences on subsequent pain scores [64,65]. One study in which mepivacaine was administered used 30 G needles [16]; however, in the five studies in which lidocaine was used, three used 25, 26, and 30 G needles, respectively, and the remaining two did not mention the needle thickness [14,39,40,44,50].

Thirdly, the observed variation may have been influenced by the size of the needle used for the RAP. Earlier reports suggested that using a smaller needle for RAP, irrespective of the presence or absence of prior local anesthesia, is linked to reduced pain [16,66]. Notably, studies using sheath introducers have been included in the RCTs that involved lidocaine infiltration. However, it would be desirable to conduct RCTs by directly comparing mepivacaine and lidocaine to ascertain whether differences in drug characteristics between these drugs influenced the analgesic effects on RAP-procedural pain.

Generalizability

Our results may provide insights that aid in the selection of more suitable local anesthesia methods, depending on the setting. We recommend employing mepivacaine infiltration or lidocaine spray in various settings, including EDs and other urgent situations (approximately five minutes or less). Mepivacaine infiltration may be advantageous in more pressing conditions (approximately one minute or less). Clinicians should take into consideration the pain associated with subcutaneous injections in cases of mepivacaine infiltration. The RCT included in this study used 1 mL of 1% (10 mg/mL) mepivacaine subcutaneously with 30 G needles [16].

If clinicians cannot utilize these options owing to allergies or other reasons, cryotherapy may be considered in time-sensitive settings. In non-urgent situations, such as scheduled blood draws for hospitalized patients or monitoring during planned surgeries, topical anesthesia alone or in combination with infiltration anesthesia is used, using available agents.

Due to the limited number of RCTs that employ sheath introducers for RAP, clinicians should be cautious when extrapolating the current results. The results of our subgroup analysis, which is limited explicitly to RAP for CAG or PCI, suggest that topical and infiltration anesthesia may be more effective than alone. Therefore, although clinicians can use mepivacaine infiltration and lidocaine spray separately, their combination may also be effective. Additional studies are necessary to establish the efficacy of the combination of mepivacaine infiltration and lidocaine spray.

Strengths and Limitations

Strengths: To the best of our knowledge, this is the first NMA to compare any local anesthetic techniques for pain associated with RAP. The primary strength of this NMA lies in its comparison of the efficacy of individual interventions rather than categories. Additionally, we differentiated between the RAP-procedural pain, encompassing local anesthesia, and the pain specifically from RAP alone. Therefore, clinicians can more easily apply the results of this study to their patients than those of the prior SR and MA [1].

Limitations: Nonetheless, this SR and NMA have some limitations. First, we limited the period of our database search to the period after the search period of the previous SR [1]; therefore, we may have missed some essential RCTs before that period. However, we attempted to overcome this limitation by conducting a citation search. Second, utilizing a higher score between the pain scores from local anesthesia and RAP may not replace the composite pain score associated with the entire RAP procedure. Although reports suggest that anxiety about future pain and past pain experiences may affect the perception of subsequent pain, the accurate assessment, overestimation, or underestimation of this influence remains controversial [67-69]. Consequently, the shape of the overall pain score for the procedure based on pain from local anesthesia and pain from RAP remains uncertain.

## Conclusions

Mepivacaine infiltration and lidocaine spray probably reduce the pain associated with RAP more than other local anesthesia. No study reported the occurrence of severe adverse events. Even in an emergency setting, such as the ED, clinicians should actively administer these local anesthetics to control pain proficiently. If they use sheath introducers, a combination of topical and infiltration anesthesia may be more effective than infiltration anesthesia alone. We anticipate that researchers will perform RCTs to compare the efficacy of mepivacaine and lidocaine infiltrations and evaluate the combination of lidocaine spray and mepivacaine infiltration against each method, mainly when using sheath introducers.
